# Legionelosis en España, 2010-2015

**DOI:** 10.7705/biomedica.5367

**Published:** 2020-09-07

**Authors:** Enrique Gea-Izquierdo

**Affiliations:** Facultad de Medicina, Pontificia Universidad Católica del Ecuador, Quito, Ecuador

**Keywords:** legionelosis/epidemiología, enfermedad respiratoria, neumonía, informes de casos, España, Legionellosis/epidemiology, respiratory tract diseases, pneumonia, case reports, Spain

## Abstract

**Introducción:**

La legionelosis es una enfermedad respiratoria bacteriana de origen ambiental que puede ser adquirida en el ámbito comunitario u hospitalario, y suele estar asociada con equipos, instalaciones y edificios. La forma clínica más conocida es la neumónica, conocida como enfermedad del legionario.

**Objetivo:**

Determinar la evolución de los casos de legionelosis en España en el periodo de 2010 a 2015.

**Materiales y métodos:**

Se hizo un estudio descriptivo de series temporales y se analizaron los casos de legionelosis notificados al Centro Nacional de Epidemiología del gobierno de España. Se determinó la distribución de los casos según el sexo, la comunidad autónoma, el mes y los grupos de edad, diferenciando en estos entre hombres y mujeres.

**Resultados:**

El recuento de casos en hombres fue superior al doble con respecto a las mujeres. La distribución en las comunidades autónomas presentó un aumento de los casos notificados al final del periodo en nueve de ellas, siendo notable en Castilla y León, Navarra y el País Vasco, y muy relevante en Castilla-La Mancha. Se estableció un patrón estacional con un pico epidémico en julio-septiembre y un mayor número de casos en torno a los 50 años de edad en ambos sexos.

**Conclusiones:**

A pesar de mostrar una prevalencia baja con respecto a otras enfermedades respiratorias, la legionelosis tiene gran impacto en la salud pública. Presenta una distribución global y heterogénea en el territorio español, con un aumento de casos en los dos últimos años, por lo que se requiere una mejor prevención y control de la enfermedad.

## Abstract (en)

**Introduction:**

Legionellosis is a bacterial respiratory disease with an environmental origin in the community or in hospitals; it is usually associated with devices, facilities, and buildings. The most common clinical form is the pneumonic, known as legionnaires’ disease.

**Objective:**

To determine the evolution of legionellosis cases in Spain from 2010 to 2015.

**Materials and methods:**

This was a descriptive study of time series with an analysis of the legionellosis cases notified to the *Centro Nacional de Epidemiología* (Government of Spain). Case distribution was determined according to sex, the autonomous community of origin, month, and age groups differentiating in the latter between men and women.

**Results:**

Case count in men was more than double compared to that in women. The cases notified by nine of the autonomous communities showed an increase at the end of the period, especially in Castilla y León, Navarra, and the Basque Country but also notable in Castilla-La Mancha. A seasonal pattern was identified with an epidemic peak in July-September and a greater number of cases among 50-years old people from both sexes.

**Conclusion:**

Despite its low prevalence compared to other respiratory diseases, legionellosis has a great impact on public health. Its distribution in Spain is global and heterogeneous with cases increasing in the last two years. Therefore, better disease prevention and control measures are recommended.

La legionelosis en una enfermedad respiratoria causada por la bacteria *Legionella* spp. cuya gravedad varía dependiendo de múltiples factores, como la edad del paciente, el tiempo de exposición a la bacteria, su concentración en el agua, el estado inmunológico del receptor y el tabaquismo, entre otros. El agente causante de la enfermedad vive preferentemente en aguas naturales y, ocasionalmente, en la tierra, contaminando así los ambientes habitados por el hombre y dispersándose fundamentalmente mediante aerosoles.

Bajo las condiciones idóneas, la bacteria causa la enfermedad en la comunidad, los hospitales o durante viajes. No se ha descrito hasta ahora el contagio de persona a persona de la enfermedad, aunque recientemente algunos autores han cuestionado el que no ocurra (1). Los grupos de edad de mayor riesgo son los mayores de 50 años; afecta en mayor medida a los pacientes inmunodeprimidos (aquellos que tienen sida, han recibido trasplante de órganos o han sido tratados con esteroides sistémicos) y a aquellos con una enfermedad crónica de base (enfermedad pulmonar obstructiva crónica, insuficiencia cardiaca congestiva o diabetes mellitus) (2-4).

La incidencia de la enfermedad varía entre países, áreas geográficas y estaciones del año, o en función de la vigilancia epidemiológica debido a las diferencias en el seguimiento y la notificación de los casos que, en ocasiones, se deben a deficiencias de los sistemas de vigilancia. A pesar de que la enfermedad tiene presencia mundial, en las zonas industrializadas es más común: en Estados Unidos, Australia y Europa llega a tasas de 10 a 15 casos por cada millón de habitantes por año (5). Hay una estrecha relación entre su incidencia y los países desarrollados, en los cuales tiene una mayor repercusión en términos de salud pública (6). En Europa, la vigilancia de la enfermedad comenzó en 1996, y la notificación es obligatoria en 30 países de la Unión Europea y el Espacio Económico Europeo. Entre el 2005 y el 2010, dicha notificación fue de 5.500 a 6.500 casos por año, con tasas anuales estandarizadas por edad de cerca de un caso por cada 100.000 habitantes (7) y con diferencias significativas entre ellos.

En España el comportamiento de la enfermedad es similar al del resto de Europa y se han descrito casos prácticamente en todo su territorio (8), con una distribución heterogénea entre comunidades autónomas. Asimismo, las tasas son mayores en hombres que en mujeres y aumentan con la edad (1). Como ocurre en Europa, la mayoría de los casos en España se presenta en el ámbito comunitario, con un incremento a partir de la década de los 90 debido, seguramente, a la detección del antígeno en la orina y la mejora en el sistema de vigilancia epidemiológica.

El objetivo del estudio fue presentar la evolución de los casos de legionelosis en España según el sexo, la comunidad autónoma y los grupos de edad, entre el 2010 y el 2015.

## Materiales y métodos

Se hizo un estudio descriptivo de series temporales en el que se analizó la información correspondiente a los casos de legionelosis registrados por el Centro Nacional de Epidemiología del Instituto de Salud Carlos III (Ministerio de Ciencia, Innovación y Universidades del gobierno de España) por medio de su Área de Análisis Epidemiológico y Situación de Salud.

En España, la enfermedad es de notificación obligatoria y los casos nuevos se recopilan semanalmente e incluyen aquellos bajo sospecha clínica. La semana es la unidad temporal básica para la notificación, la agregación de los datos y su análisis. Para el registro de la información, se considera que cada semana se acaba a las veinticuatro horas del sábado y los datos correspondientes deben remitirse el siguiente lunes. Estos se envían como datos numéricos desagregados de los estratos inferiores inmediatos y, también, incluyen el total del nivel superior que informa. La recolección y el análisis de la información epidemiológica se hace por medio de la red nacional de vigilancia epidemiológica, con el fin de detectar problemas, valorar los cambios en el tiempo y en el espacio y contribuir a la aplicación de medidas de control individual y colectivo de las condiciones que supongan un riesgo para la salud por su incidencia nacional o internacional, así como para difundir la información en los niveles operativos competentes. Esta red se encuentra al servicio del Sistema Nacional de Salud de España (9).

En este estudio, se incluyeron los casos registrados entre el 2010 y el 2015 con diagnóstico principal de legionelosis confirmada y se excluyeron los pacientes con datos incompletos o que no cumplían con los criterios clínicos y paraclínicos de legionelosis. Se analizaron las siguientes variables: sexo, edad, comunidad autónoma de origen, año, mes, especie, serogrupo, ámbito, categoría de casos y método principal de diagnosis (Estas variables no aparecen en el texto.).

La distribución de los casos se analizó según el sexo, la comunidad autónoma de origen, el mes y los grupos de edad, diferenciando en ellos entre hombres y mujeres. La asociación entre el mes en que se notificaron los casos y el año, y aquella entre el grupo de edad y el sexo, se determinaron mediante una prueba de ji al cuadrado (p<0,05: estadísticamente significativo). En el análisis estadístico se empleó el programa Stata 14.2™ (Statistics/Data Analysis, Special Edition, StataCorp LLC, Texas, USA).

## Resultados

**Figura 1:**
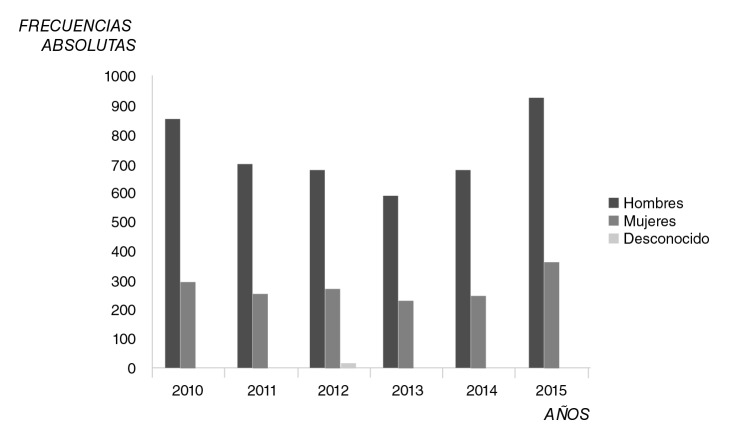
Casos notificados de legionelosis según sexo, España, 2010-2015

Según el sexo, el número de casos de legionelosis notificados en España entre el 2010 y el 2015 fue mayor entre los hombres (frecuencias absolutas), con el mayor número de casos (n=932) en el 2015, año en que también se dio el mayor número de casos en mujeres (n=366) (figura 1). En general, el recuento de casos en hombres fue más del doble que en mujeres y en ambos sexos se apreció un “valle” en la evolución de los casos desde el inicio de la serie hasta el final.

Por otro lado, con una representación mucho menor con respecto a los hombres y mujeres, el número de casos desconocidos supuso valores prácticamente irrelevantes en los años 2010, 2011 y 2013 e inexistentes en el 2014 y el 2015, en tanto que en el 2012 se registraron 19 casos.

**Figura 2:**
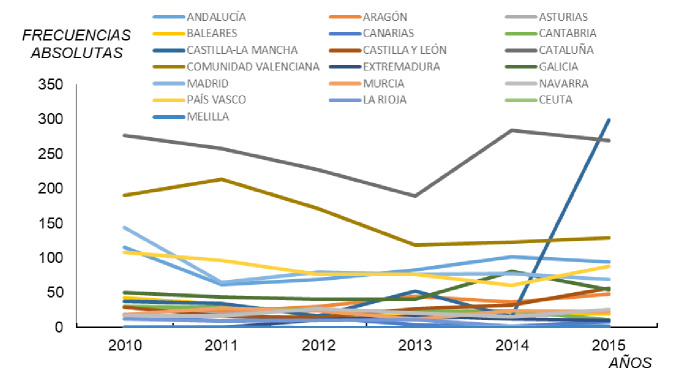
Casos notificados de legionelosis según comunidad autónoma, España, 2010-2015

En cuanto a los casos notificados según la comunidad autónoma de origen, durante el periodo de estudio, en la mayoría de las comunidades hubo un promedio anual inferior a los 100 casos (figura 2) No se entiende la figura 2, pues hay ¡19 variables representadas en líneas! Sin embargo, hay que señalar que, en Castilla-La Mancha en el 2015, se registró un pico epidémico con 299 casos frente a los 15 del 2014, lo que llama la atención, sobre todo porque fue el mayor número de casos en todo el periodo. También Cataluña y la Comunidad Valenciana, especialmente la primera, presentaron cifras altas, lo que respondería a muy diversos factores como grado de desarrollo industrial, actividad turística, comunicación de los casos, aspectos medioambientales, condiciones hospitalarias, etc.

En general, se podría afirmar que hubo un umbral de casos en la mayoría de las comunidades autónomas, así como similitud dentro de la serie y ausencia de cambios apreciables en la tendencia. Solo se destacaron el 2013, con 52 casos en Castilla-La Mancha, y el 2014, con 81 en Galicia y con 102 en Andalucía. En este último año, Cataluña presentó 284 casos. En la figura 2 se aprecia el aumento de casos notificados en el último año, con respecto al promedio en los dos años anteriores.

**Figura 3:**
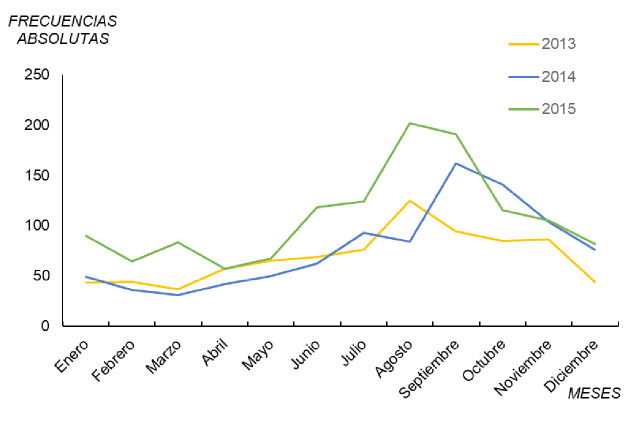
Casos notificados de legionelosis por mes, España, 2013-2015

Entre el 2013 y el 2015, se constata la periodicidad en el aumento de casos notificados según el mes (figura 3). De hecho, es un patrón estacional (finales de primavera-verano) que se repitió en la mayoría de los años, con un pico epidémico al final del verano (julio-septiembre) y un descenso en los meses más fríos, en tanto que en la primavera se registró un aumento paulatino del número de casos registrados.

En la figura 3, se observa claramente el aumento del número total anual de casos de legionelosis en el 2013, el 2014 y el 2015, con 825, 930 y 1.298 casos. En los meses de junio, julio y agosto, prácticamente se registró el doble de casos entre el 2013 y el 2015. Por último, cabe mencionar que, en el 2015 hubo una tendencia atípica con respecto a los otros dos años en el periodo de enero a marzo, así como valores más altos en el último mes del primer trimestre del año, comportamiento anual que podría deberse a factores meteorológicos

**Figura 4:**
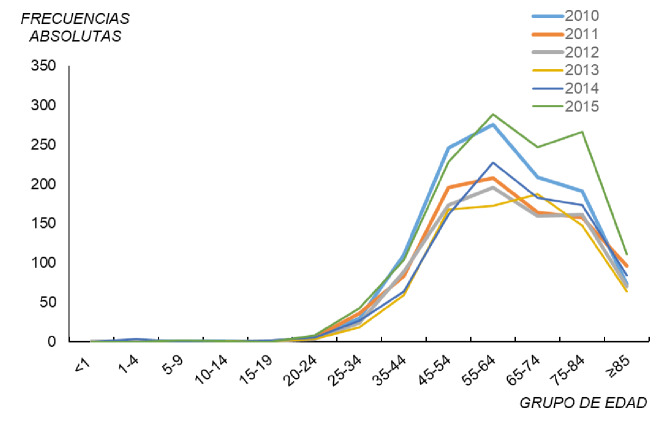
Casos notificados de legionelosis por grupo de edad en ambos sexos, España, 2010-2015

En lo que respecta a la notificación por meses y años (2013-2015), no hubo asociación (χ^2^=36; p=0,33), como tampoco la hubo entre el trimestre y el año (χ^2^=4; p=0,26). En el periodo de estudio, la tendencia de los casos notificados de legionelosis en ambos sexos según los grupos de edad indica que, en general, a partir del grupo etario de 20 a 24 años y hasta el intervalo de 55 a 64 años, esta fue al incremento (figura 4). Después de estas edades, hasta los 85 años o más, y salvo excepciones (2012, 2013 y 2015), se produjo un descenso en las frecuencias absolutas. Las excepciones se dieron en los intervalos de 75 a 84, 65 a 74 y 75 a 84 años de edad. Debe señalarse que, en el 2015 y el 2010 (series superiores), se registró el mayor número de casos.

Puede afirmarse que hubo más casos en personas de edad avanzada de ambos sexos, aunque sin desestimar el impacto en otros grupos de edad. Ello explica que no se haya encontrado asociación entre el grupo de edad en ambos sexos y el año (χ^2^=13; p=0,37).

**Figura 5:**
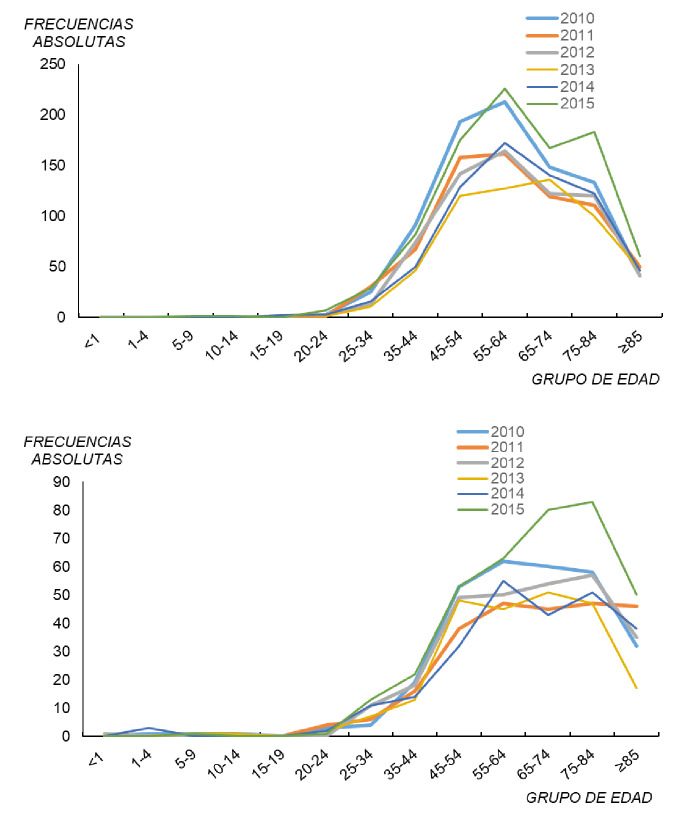
Casos notificados de legionelosis por grupo de edad en hombres y mujeres, España, 2010-2015

En los hombres, el comportamiento en las tendencias de la serie (figura 5) fue muy similar a los casos notificados de legionelosis por grupo de edad en ambos sexos. Únicamente, en los años 2013 y 2015 se produjo una ruptura en el declive de los casos desde el grupo 55-64 (65-74 y 75-84, respectivamente). En el 2010 y el 2011, no se notificaron casos en los grupos de edad de menores de 20 a 24 años, en el 2012 y el 2013, solo hubo un caso en el grupo de 15 a 19 y dos en el 2014, y en el 2015, se registró un caso en el grupo de 5 a 9 años y otro en el de 10 a 14. Hay que resaltar que la legionelosis afectó más a los hombres que a las mujeres, lo que podría deberse a factores laborales, al tabaquismo, a antecedentes de otras enfermedades, etc. 

En España, los casos notificados de mujeres fueron menos que los de hombres, con una variación en cuanto a los grupos de edad en los que la enfermedad tuvo más presencia, ya que, aunque el grupo de edad de 55 a 64 años siguió siendo el más afectado, también entre las personas entre los 75 y los 84 años hubo tasas importantes en el 2012, el 2014 y el 2015 (figura 5). En el 2011, el número de casos se estabilizó en los grupos de edad de 55 a 64 años en adelante y, al igual que en los hombres, el 2015 y el 2010 presentaron el mayor número de casos. Por debajo del grupo etario de 20 a 24 años, se notificaron muy pocos casos, excepto en el 2011 cuando no hubo ninguno, en tanto que en el 2014 hubo tres casos en el grupo de edad de 1 a 4 años.

## Discusión

En términos generales, la salud exige una perspectiva intersectorial que garantice su promoción y la prevención de enfermedades para el mejoramiento de la calidad de vida de las personas. La neumonía constituye hoy un objetivo prioritario de salud a nivel mundial, pero persisten deficiencias en la determinación de los agentes que la propician. En ciertos países, ello puede deberse a la falta de recursos económicos, al desconocimiento de la metodología de muestreo e identificación de microorganismos, al diagnóstico insuficiente, a las deficiencias en el funcionamiento del sistema de vigilancia epidemiológica, etc.

En cuanto a la epidemiología de la enfermedad, todavía queda mucho por determinar. Para aportar información específica útil para la vigilancia epidemiológica, los hallazgos microbiológicos confirmados por el laboratorio permiten detectar la circulación de los diferentes agentes etiológicos, sus características y sus patrones de presentación y, por el otro, se caracterizan los brotes epidémicos. Además, se caracterizan los brotes epidémicos, identificando nuevos agentes y enfermedades emergentes mediante novedosos elementos de vigilancia que permiten detectar la resistencia a los antibacterianos, así como marcadores epidemiológicos.

En el 2014, el número de defunciones por neumonía en la Unión Europea fue de 120.000, aproximadamente, siendo mayor en las mujeres que en los hombres en la franja de edad de 65 años y más. Podría afirmarse, entonces, que la neumonía es una de las enfermedades respiratorias más frecuentes y más letales; en ese contexto, España ocupa el sexto lugar, por detrás de Reino Unido, Alemania, Polonia, Francia e Italia, con cerca de 10.000 muertes al año. No obstante, el aumento de la esperanza de vida, así como una mayor supervivencia de personas con enfermedades crónicas o deficiencias inmunológicas, hace pensar que la enfermedad podría haber modificado su comportamiento en los últimos años. De hecho, en el 2016 España fue el tercer país de la Unión Europea con más casos de neumonía (10), por detrás de Reino Unido y Francia; aunque presenta cifras inferiores a la media europea, ¿esto no ocurre en menos de un año?. En este sentido, en años recientes se ha hecho un gran esfuerzo en la comprensión de la etiología de la enfermedad para un mejor diagnóstico; además, el impulso a estrategias terapéuticas novedosas y un mayor conocimiento de la resistencia bacteriana, constituyen una esperanza en el combate contra la enfermedad.

En cuanto a la legionelosis, el número de casos notificados en Europa se mantuvo estable en la última década, aunque hubo un pico en el 2010, y posteriormente se regresó a los valores registrados entre el 2005 y el 2009. En los últimos años (2014-2015) se han detectado las tasas más altas de notificación de casos (11), lo que podría deberse a factores, como una mayor vigilancia epidemiológica, mejores notificaciones (12), el clima (lluvia, temperatura) y la mayor precisión en el diagnóstico. Además, cabría pensar que el aumento que se está produciendo en Europa en los mayores de 65 años podría contribuir al incremento del número de casos, ya que estos presentan mayor riesgo de contraer la neumonía. (Verificar el significado con el autor. El original era muy confuso. Nota: se habla aquí de neumonía pero el párrafo alude a la legionelosis.)

Entre el 2011 y el 2015, España, Francia, Alemania e Italia notificaron el 70,3% del total de los casos europeos, en una población que representaba solo el 49,9% del total considerado (13). El ámbito preferente fue el comunitario y, al igual que la neumonía, afectó en mayor medida a los hombres que a las mujeres y a las personas de edades avanzadas (cerca de 80% de los casos en mayores de 50 años). En España, el impacto de la legionelosis también fue más patente en hombres que en mujeres, como ocurre con la neumonía, siendo mayor en la última. (Esto ya estaría implícito en la frase anterior, puesto que España está entre los países mencionados.) Además, la presencia de la legionelosis es particularmente importante en el medio hospitalario y entre los turistas, tanto en quienes contraen la enfermedad en España como en los casos de españoles que enferman en el extranjero. Curiosamente, la neumonía, que en el ámbito hospitalario no supera el 2%, puede llegar a alcanzar el 24% en pacientes hospitalarios e incluso superar el 40% en enfermos de cuidados intensivos (¿Afirmación contradictoria? ¿No supera el 2%, pero luego se habla del 24 y el 40%?) (14). Por su parte la legionelosis puede alcanzar una mortalidad entre el 10 y el 30%, desde un 3% en la comunidad hasta un 30% en hospitales (15). Por esto, cabría plantear la posibilidad de ejercer un mayor control de los brotes en el ámbito hospitalario, más que en el comunitario (16).

En este sentido, debe señalarse que la información epidemiológica que permite identificar la posible fuente de infección es de gran importancia; no obstante, al correlacionar la evolución de las normas para prevenir la legionelosis, con los casos registrados y la mortalidad, puede afirmarse que la implementación nacional de dichas normas sanitarias no está siendo suficientemente eficaz en el control de la enfermedad. En realidad, sería más preciso afirmar que la aplicación de las normas está más dirigida al número de casos que a las defunciones, lo que podría deberse a que el primero es una expresión directa, ¿en tanto que la segunda podría aumentar sin grandes diferencias ante un número constante de casos?

Además, la mortalidad puede estar supeditada a la correcta identificación del microorganismo y a factores relacionados con el tratamiento y otros no implicados en la prevención meramente normativa. Asimismo, el aumento en los casos notificados puede deberse a desajustes en el cumplimiento de las normas, a una mejor notificación como consecuencia de una adecuada operación del sistema de vigilancia epidemiológica, o a otros factores externos.

Estos factores, no descritos o estudiados en profundidad en la literatura científica, podrían resultar básicos para explicar ciertos comportamientos epidemiológicos que no se han comprendido del todo e, inclusive, como herramientas para un mejor control de la enfermedad. La determinación de tales factores permitiría predecir temporalmente fenómenos epidemiológicos y un uso adecuado de los recursos (17). La vigilancia epidemiológica y el estudio de los factores asociados podrían explicar la concentración de la notificación de brotes en determinadas comunidades autónomas, áreas o ciertos meses del año. En otras ocasiones, la confluencia de eventos en ciertas regiones podría deberse a otros factores específicos de ellas, como pueden ser los cambios ambientales estacionarios, por ejemplo, una ola de calor combinada con abundantes lluvias (18). De hecho, es objeto de debate el impacto del clima sobre la presencia, desarrollo y dispersión de *Legionella* spp. y, por ende, su posible influencia en los casos notificados (por ejemplo, la estacionalidad detectada en la serie española de 2013 a 2015 en julio a septiembre) (19). Sería razonable vincular aspectos geográficos nacionales con el posible impacto climatológico para explicar la incidencia de la enfermedad en el territorio español.

Además, cabe pensar que la variación estacional inherente a los registros puede tener un carácter propio o global (20). Este último se hizo patente en el 2010, cuando se presentó un verano especialmente cálido con el consecuente aumento de los casos registrados, hecho que afectó fundamentalmente a Francia, Alemania y Holanda, entre agosto y septiembre (21,22). No obstante, en Europa, incluido España, sería necesario recopilar más información sobre variables medioambientales que pudieran ayudar a explicar si determinadas zonas están más afectadas por la enfermedad debido a la influencia climática, especialmente considerando que los últimos cinco años han sido los más calurosos de la historia (el 2019 fue el año más cálido registrado en Europa, incluyendo todas las estaciones) y el segundo más cálido a nivel mundial (23).

Así, la precipitación (24), la temperatura, la insolación, la presencia o ausencia de viento, su dirección, etc., podrían ser variables cuyo estudio aportaría al sistema de vigilancia epidemiológica y serviría para comparar áreas, regiones, provincias o comunidades autónomas a la hora de explicar brotes o eventos de interés. Quizá, la legionelosis, que mata aproximadamente a 500 personas por año en la Unión Europea y registra una tendencia a aumentar desde el 2005, podría limitarse a determinadas áreas geográficas y a ciertos periodos del año a la luz de los factores contemplados.

De hecho, los últimos cinco años (2015-2019) han sido los de mayor temperatura media desde que se tienen registros, con una temperatura global de 0,2 ^o^C desde el 2015, superior a lo reportado entre el 2011 y el 2015. En Europa fue particularmente caluroso el mes de julio de 2019, cuando alcanzó máximos históricos (25). Cabe mencionar, asimismo, que ciertas variaciones epidemiológicas pueden deberse a cambios demográficos o a que los periodos estudiados no sean lo suficientemente largos como para establecer el posible efecto del envejecimiento de la población. Así, la notificación de casos de legionelosis puede estar relacionada con un incremento en el número de personas de edad avanzada en riesgo (26). Por el contrario, en España no puede asumirse que la disminución en los registros se deba a mejoras en las técnicas de identificación de los laboratorios homologados para tal fin, o a inconformidades en el test de diagnóstico o, incluso,a errores en los requisitos microbiológicos.

Por último, las diferencias establecidas según los grupos de edad podrían asociarse con factores de comportamiento como el tabaquismo, que presenta el mayor número de casos en personas alrededor de los 50 años de edad. Si bien es cierto que históricamente este factor ha estado presente más en los hombres que en las mujeres, en los últimos años ha disminuido la prevalencia en ambos sexos, aunque de forma más acusada en los hombres. Para un mejor control del seguimiento de este factor y su relación con la enfermedad, sería preciso el seguimiento de cohortes de afectados y este es un vacío en el estudio de la enfermedad, ya que la recolección de datos sobre ciertos factores de riesgo (27) o comorbilidades asociadas con la legionelosis no se hace de rutina. Quizá algunos de estos factores podrían ayudar a explicar las ocasionales variaciones entre sexos o grupos de edad (28,29). Sería oportuno, además, estimar los factores sociales determinantes de la enfermedad, así como la actividad profesional de los pacientes; en este sentido se lograría una mayor precisión si la vigilancia epidemiológica contemplara las características demográficas, la localización exhaustiva de las fuentes de infección y el conocimiento adecuado de los test de laboratorio.

En definitiva, aunque pareciera que la legionelosis no tiene una gran repercusión sanitaria, no hay nada más alejado de la realidad, especialmente porque existe la posibilidad de brotes ocasionados por la falta de prevención de la enfermedad. Por ello, no debe permitirse una inadecuada vigilancia epidemiológica ni una deficiente aplicación de los mecanismos de control higiénico-sanitarios orientados a combatir el agente inductor de esta enfermedad.
